# Health care manager commitment towards implementation of citizen charter standards and associated factors in public hospitals of Jimma zone, Southwest Ethiopia

**DOI:** 10.1186/s12913-021-07137-1

**Published:** 2021-10-23

**Authors:** Nigusu Getachew, Gebeyehu Tsega, Firehiwot Worku, Tilahun Fufa Debela, Dejene Melese, Elias Ali Yesuf, Yibeltal Siraneh

**Affiliations:** 1 Department of Health Policy and Management, Faculty of Public Health, Institute of Health, Jimma University, Jimma, Ethiopia; 2 Department of Public Health, School of Public Health, College of Medicine and Health Science, Bahir Dar University, Bahir Dar, Ethiopia

**Keywords:** Healthcare manager, Commitment, Associated factors, Implementing citizen charter, Jimma zone

## Abstract

**Background:**

Managerial commitment is important for effective design and implementation of citizen charter to assure the quality of health service delivery as per the standards depicted in the document. Hence the objective of this study is to assess the level of managerial commitment towards implementation of the citizen charter standards and associated factor in Jimma zone public hospitals.

**Methods:**

A Facility based cross-sectional study design was employed in Jimma zone public hospitals from March 14 to May 16, 2019 and 422 health managers who were currently working at all levels of management were participated in the study. After checking its completeness, the data was entered into EPI data version 3.1 and exported to SPSS version 20 for statistical analysis. Factor analysis was conducted. Simple and multiple linear regression were done using 95%CI and significance was declared at *P* < 0.05. All assumptions of linear regression and principal component analysis were checked.

**Results:**

The percentages mean score of managerial commitment for health managers working in jimma zone public hospitals was 58%. Perceived value and care for managers (β = .329,95% CI,.245,.413, *p*-value<.001), Interaction between staff and managers’ (β = 0.077,95%CI,.032,.122, *p*-value< 001),involvement during implementation of citizen charter(β = 0.061,95%CI,.010,.112,*p*-value = 018) and positional level(β = − 122,95%CI,-.242,-.002,p-value = .046) as predictors of managerial commitment towards implementation of citizen charter standards.

**Conclusions:**

In this study, the percentage mean score of managerial commitment for health care managers working in Jimma zone public hospitals was medium. Hence, all levels of managers to consider and maintain factors identified in this study in their management practice to foster a higher level of managerial commitment towards implementation of citizen charter standards in jimma zone public hospitals.

## Background

Citizen charter (CC) is a document that an organization publishes for public reference, and which provides details about the nature, working, and functions of the organization. A CC represents the commitment of the Organization towards standard, quality and time frame of service delivery, grievance redress mechanism, transparency and accountability. CC ensures that public institution dealing with service delivery does so in efficient, effective and timely manner. It creates awareness about citizen’s rights, enhances accountability of the organization, and improves service delivery [1] .

The CC has been developed as a tool to improve the quality of services, address the needs of citizens’ rights and set clear standards of performance because bureaucratic administration is regarded as rigid, rule bound, slow moving, costly, inefficient and unresponsive to them users [2] . It was introduced first in federal ministry republic of Ethiopia, ministry of civil service in February 2012. The other government organization also agreed to develop their own charter. Researches are encouraged to explore the contribution of CC in improving public service delivery and accountability at the local administration level [3].

CC standard was introduced in Jimma zone Hospitals in 2016, where the public bodies have been able to improve their functioning over the years by referring to the principles and time lines enshrined through these charters, thus promoting good governance, and greater satisfaction of the citizens in general. The charter enables the service seekers to avail the services of the government departments with minimum inconvenience and maximum speed. For this, the CC are expected to indicate ‘WHERE TO GO’ and ‘HOW TO PROCEED.’ On the other hand, it makes the service providers aware of their duties to attend to the problems of the concerned citizens within a reasonable time-frame [1].

Managerial commitment is important for the implementation of CC standards to the interest of the public service provision and institutionalization of the elements of efficiency, accountability and transparency in the public service. The managers should play a crucial role in guiding the organization and its policies in order to assure the quality of service delivery for the clients. Moreover, managers act as an engine of inspiration for the fellow staff members so as to realizing the organization’s vision and objectives. For instance, by giving reward for outstanding employees in order to acknowledge the contribution they made to the organizations [4].

As Samia Javed argued that the managers should not only concern for performing the office tasks rather they have conduct observation the all over application of the organization with the particular aim of identifying the problem of the customers and the service providers. Also they shouldn’t monopolize the decision making process rather they should late this power to the lower level managers and the senior managers should focus only on reviewing the lower manager’s decision [5] . A research conducted in Vietnam and Bangladesh revealed that some of sociodemographic characteristics (age and marriage),job satisfaction components and perceived organizational support have a significant impact in an individual commitment levels to implementation of citizen charter [6–8] .

The achievement of quality in service provision is not an immediate action rather it realizes progressively. These progressive developments presuppose the proper designation and implementation of citizen charter. It has a number of advantages for the customers and providers including improving quality of service delivery, enhancing accountability, minimizing corruption, and tracking service delivery performance. The presence giving priority for the customer, cooperation between staff and management and a well-functioning grievance redress mechanism will bring effective implementation of citizen charter. By maintaining a commitment to continuous improvement, task teams can help service providers design and implement citizen charters that promote better development outcomes [9]. It is imperative to know the level of health care manager commitment towards implementation of citizen charter standards in the health care setting of Ethiopia that helps to understand the implementation of citizen charter standards. Therefore, this study is conducted to assess the level of healthcare manager commitment towards implementation of the citizens ‘charter standards and associated factor particularly in the hospitals of Jimma zone to improve the quality of the services.

## Methods

### Study design, setting and period

A facility based cross sectional study design was conducted in Jimma Zone Hospitals southwest of Ethiopia from March 14 to May 16, 2019. The zone has eight governmental hospitals, two private hospitals, and 120 health centers. Out of the eight governmental hospitals, one is a referral hospital Jimma University Medical Center (JUMC), three are general hospitals and four are primary hospitals. It provides service for a total population of 2,486,155 out of which 1,250,527 are male (Source HR, Plan, and program office).

### Population

The source and study population were all level health care managers and selected healthcare managers working in Jimma zone hospitals respectively. All level managers who worked for six months and above on position in the zone were included and managers who were delegated during data collection were excluded in the study.

### Sample size determination and sampling procedure

Single population formula was used assuming 95% confidence interval and 50% prevalence (P) due to lack of such study, and a precision of 5% between the sample and the 10% non- response rate is taken, thus a total of 422 health managers required for the study. Simple random sampling techniques were used to select the hospitals. Form the total of ten hospitals found in the Jimma zone, four (Jimma University medical center, Agaro general hospital, Seka primary hospital, and Limu general hospital) were selected for the study and 169 all level managers from JUMC, 87 from Agaro hospital, 79 from Seka, and 87 from Limmu hospital. Finally, proportionally allocate to each hospital then, all eligible study participants were selected from each hospital till the allocated sample size reach.

### Study variables

The dependent variable was health manager commitment and the independent variables were socio-demographics, job satisfaction, perceived organizational support and perceived personal characteristics.

### Data collection instrument

The data were collected using self-administered, structured and pre-tested questionnaire that was adapted from different literatures and translated into Amharic then back into English [6, 8–10]. The tool contains five parts and has 75 items. Part 1 is on socio-demographic and economic data that comprise 9 items. Part two has 26 items of 5-point Likert scale to measure job satisfaction with. Part 3 contains 20 items of 5-point Likert scale to measure perceived organizational support. Part 4 comprises of 10 items with single component that measure managerial commitment and part five is made up of 10 items. Under each dimension, higher score indicates higher perception towards the emerged scale.

### Data processing and analysis

The completed questionnaire was coded and entered a data entry template in EPI-DATA version 3.1 then exported to SPSS version 20 for analysis. Descriptive statistics like raw means, standard deviations, mean scores, and summary tables was used for describing the data. The negatively worded items were reverse-coded. All assumptions of linear regression and PCA were checked. Multicollinearity was checked by examining the variance inflation factor. Varimax rotation was employed during factor extraction. Simple linear regression was conducted and significant variables at *p*-value< 0.25 were taken as candidate for hierarchical multiple linear regressions. Factors predicting Managerial commitment were identified using multiple linear regression analysis at a significance level of *p*-value < 0.05 with 95% confidence interval. The reduced final model was constructed using backward model selection method. Split sample validation and outliers’ detection were done using principal component analysis. After conducting factor analysis, the name given for each latent component (factor with Eigen value greater than 1 extracted), and the emerged latent variables were perceived organizational rules and regulation scale, perceived value and care for manager scale, perceived concern for employee immediate manager scale, Involvement during development of citizen charter scale, communication and adequate staff scale, perceived promotion and recognition scale, Perceived remuneration scale, Interaction between staff and managers’ scale, perceived autonomy at work scale, Perceived working environment scale, I’m responsive and client centered in my position scale, I’m being responsible and accountable for my action scale, and managerial commitment scale and the items with scale of reliability coefficient (Cronbach’s alpha) of greater than 0.70 were considered. Only items having a communality of > 0.50 on factor analysis were retained for further analysis.

### Ethics approval and consent to participate

Ethical clearance was taken from Institutional Review Board of Jimma University Institute of health, and permission letter was obtained from zonal health department including from the respective hospital management. Written informed consent was obtained from all participates prior to their participation with informing the purpose, benefits, confidentiality of the information, and voluntary nature of participation in the study. Name and other personal identifiers were not recorded to maintain confidentiality. “All the protocol was performed in accordance with the relevant guideline and regulation.”

## Results

### Socio-demographic characteristics

Out of 422 health managers, 420(99.5%) were returned the questionnaires, making the response rate of 99.5% and majority of the participants were males 326 (77.6%). The mean age was 32.4 ± 5.02 years. More than two third of the participants were married 298 (70.9%). The mean work experience at the current hospital was 5 ± 3.98 years, more than half 245(58.4%) of them has bachelor’s degree level of educational qualification). Three hundred thirty one (78.8%) of the participants were working in a lower level managerial position (Table 1).

**Table 1:** Frequency distribution of socio-demographic characteristics of health care managers working in Jimma zone hospitals, southwest, Ethiopia, 2019(*n* = 420)

Variable	Category	No (%)
Age	26–30	171 (40.7)
	31–35	156(37.1)
	36–40	56 (13.3)
	> = 41	37 (8.8)
Gender	Female	94 (22.4)
	Male	326(77.6)
Marital status	Single	122 (29.1)
	Married	298(70.9)
Profession	Medical doctor	73 (17.4)
	Nurse all type	236 (56.1)
	Midwife	26 (6.2)
	Medical laboratory	24 (5.7)
	Pharmacy	22(5.2)
	Other*	39 (9.3)
Work experience	<=2	21 (5)
	2.01–5	265 (63.1)
	5.01–10	103(24.5)
	> 10	31 (7.4)
Qualification	Specialist	25 (5.9)
	Master’s degree	36 (8.6)
	Bachelor degree	277(66)
	Diploma	47 (11.2)
	Certificate	35(8.3)
Positional level	Top level	18 (4.3)
	Middle level	95 (22.6)
	Lower level	307 (73.1)

### Level of health care manager commitment

The percentage mean score of healthcare managers who participated in this study was 58%(SD10.99). On the other hand, mean raw score of this scale was 32.83 ± 4.4 with total rotated variance explained 82.81% using principal component factor extraction analysis.

### Level of perceived organizational support

Perceived communication and adequate staff were the highest 57.7% followed by perceived concern for employee immediate manager scale was 46.6%. The higher score indicates higher perceived organizational support. The total variance explained by the model 67.7%.(Table 2).

**Table 2:** The mean score of level of perceived organizational support of managers working Jimma zone hospitals, southwest, Ethiopia, 2019

Emerged factor (scale)	Mean raw score ± SD	SM%
Perceived organizational rules and regulation	17.4 ± 6.3	36.7
Perceived value and care for manager	10.9 ± 3.2	43.2
Perceived concern for employee immediate manager	8.6 ± 2.1	46.6
Involvement during development of citizen charter	8.3 ± 2.3	42.6
Perceived communication and adequate staff	9.9 ± 2.4	57.7

#### Level of perceived job satisfaction

Perceived staff and managers’ interaction was the highest 58.5% followed by Perceived remuneration scale was (53.20%). The higher mean scores indicate higher job satisfaction in relation with these variables. All the extracted, explain a total variability of 70.26% resulted from rotated total variance explained. (Table 3).

**Table 3:** Mean score for perceived job satisfaction of managers working in, Jimma zone hospitals, southwest, Ethiopia, 2019

Emerged factor (scale)	Mean raw score ± SD	SM%
Perceived recognition and promotion	19.6 ± 7.9	33.8
Perceived Autonomy at work	9.4 ± 3.3	35.2
Perceived Remuneration	18.8 ± 6.3	53.2
Interaction between staff and managers’	13.4 ± 3.4	58.5
Perceived availability of resource and working environment	7.7 ± 2.7	39.5

%SM is the Standardized mean score as the percentage of possible maximum scale score, and it lies between 0 and 100

#### Level of perceived personal characteristics

Perceived Personal characteristics were ‘percentages mean score of manager of 70.8%. The higher score indicates higher perceived personal characteristics, participants had in line with expected administrative ethical practice. The mean raw score was 38.3 ± 5.1 with a total variance explained by the model 66.93%.

### Independent predictors of health manager commitment

Those variables which had statistically significant association with health managers’ commitment in the preceding four models were entered into the final model (MLR). This model explained almost 94% (R = 0.969, R-Square = 0.940, Adjusted R Square = 0.935) the variability in the managers commitment. None of perceived personal characteristics variables were significant predictor in the final model. (Table 4).

**Table 4:** Independent predictors of healthcare manager commitment of managers working in, Jimma zone hospitals, southwest, Ethiopia, 2019

Variables	Unstandardized Coefficients	Standardized Coefficients	Sig.	95% CI
	B	Beta		
Perceived Value and care for manager	.329	.329	.000**	(.245,.413)
Involvement during development of the Citizen Charter standard	.061	.061	.018*	(.010,.112)
Gender (female were reference)	−1.183	.593	.0001**	(− 1.329, − 1.036)
Interaction between staff and managers’	.077	.077	.001*	(.032,.122)
Ward/department	−.154	−.042	.072	(−.322,.014)
Positional level **(**Middle level managers were reference)	−122	.048	.046*	(−.242, −.002)
Age	−.102	−.091	.036*	(−.196, −.007)

Constant .849, R = 0.969, R Square = 0.940, Adjusted R Square = 0.935, * significant at *p* value < 0.01 **significant at p value < 0.001, Max VIF = 3.8 < 5VIF

In this study a one-unit increment in interaction between staff and managers’ score resulted in 0.077-unit increase in the managers commitment score of managers (β = 0.077, 95%CI: .032.122, *P* = .001).

One-unit increment in the involvement during development of the Citizen Charter standard factor score of the managers increases their managerial commitment score by 0.061 (β = 0.061, 95%CI:.010,.112 *p* = 0.018).

A unit increment in age for manager lead to a decrement of managerial commitment score by 0.102 (β = −.102, 95%CI −.196, −.007, *p* = .036).

The study showed negative predictive relationship between positional level of managers’ and managerial commitment (middle level managers were reference). Middle level managers increase commitment by 0.122 than lower level managers (β = −.122, 95%CI-.242, −.002 *p* = .046).

It was found that a unit increment in the perceived value and care for manager score leads to an increment of managerial commitment score by 0.329(β = 0.329, 95%CI, 0.245, 0.413, *p* < 001).

## Discussion

Our study pointed out that health manager commitment percentage mean score of all managers participated in this study was 58%. In this study managers might be challenged with different factor like organizational culture, belief, and values in individuals and in the organization shared which a key to introduce new system and effectively implement it. The citizen charter is powerful tool for service delivery if any level of manager is committed to implement it in a proper way. Commitment at top management level is critical and will be a strong motivating factor. This was compelling all members of staff to work as a team in achieving the organizational vision and objective. However, the introduction and implementation process have its own challenges [9].

In case of Jimma zone hospitals most of the time, the top level managers were not available in the center because they go to other place and even out of the country for meeting and training; this probably has its own impact on their commitment towards implementing the citizen charter. As the result indicated that middle level managers had better commitment than the others, this probably imply that they might have better attitude and motivation than lower level managers and also they might have more time than top level managers.

In this study, from job satisfaction related factors, Interaction between staff and managers’ score was the predictor of managerial commitment. This finding was somewhat similar with the finding of previous studies that showed some of the components of job satisfaction like staff and nurse manager interaction were influential in explaining managerial commitment of managers [11].

In addition, this finding was also consistent with study that showed positive association between staffing and managerial commitment [12]. These imply that managers who were satisfied with the communication and interaction with staffs and the support they get from their staffs were more likely to be committed to their position as well as to the organization than their counterpart. In fact, people who have higher job satisfaction are more loyal to their employer and committed to their job. Therefore, they can satisfy their needs and have positive feelings towards the position and other staffs to interact in a good way [13].

Manager’s commitment was positively associated with job satisfaction of managers that is consistent with Locke, Job satisfaction which is a pleasurable or positive emotional feeling resulting from one’s evaluation towards his/her job when comparing between what he/she expects and what he/she actually gains from his/her job [14–16].

### Limitation of the study

Finally, it must be noted that there were some limitations of the study were information bias like social desirability bias. The tool was comprised of both positive and negative statements that enhance their concentration to minimize their biased response. Difficult to name the factor on split loading effect.

## Conclusion

The level of health manager’s commitment was medium affected by age, gender, positional level, Interaction between staff and managers’, perceived value and care for manager and involvement during the implementation of citizen charter were a predictor of managerial commitment. Hence, all levels of health managers to consider and maintain factors identified in this study in their management practice to foster a higher level of managerial commitment towards implementation of citizen charter standards in the hospitals.

## Data Availability

Data will be available upon request from the corresponding author.

## Abbreviations

CC: Citizen Charter
POS: Perceived Organizational Support
SPSS: Statistical Package for Social Studies
SM%: percentage of maximum possible scale score
VIF: Variance Inflation Factor
